# Therapeutic Cerebral Fluid Puncture in Patients with Idiopathic Intracranial Hypertension: No Short-Term Effect on Neurocognitive Function

**DOI:** 10.3390/brainsci14090877

**Published:** 2024-08-29

**Authors:** Cem Thunstedt, Dilan Aydemir, Julian Conrad, Elisabeth Wlasich, Sandra V. Loosli, Florian Schöberl, Andreas Straube, Ozan E. Eren

**Affiliations:** 1 Department of Neurology, LMU University Hospital, LMU Munich, 81377 Munich, Germany; dilan.aydemir@hotmail.com (D.A.); julian.conrad@umm.de (J.C.); elisabeth.wlasich@med.uni-muenchen.de (E.W.); sandra.loosli@med.uni-muenchen.de (S.V.L.); florian.schoeberl@med.uni-muenchen.de (F.S.); andreas.straube@med.uni-muenchen.de (A.S.); ozan.eren@med.uni-muenchen.de (O.E.E.); 2 Division of Neurodegenerative diseases, Department of Neurology, Universitaetsmedizin Mannheim, University of Heidelberg, 68167 Heidelberg, Germany; 3 Department of Neurology, University Hospital and University of Zurich, 8091 Zurich, Switzerland; 4 Department of Neurology, Bogenhausen, Munich Hospital, 81925 Munich, Germany

**Keywords:** idiopathic intracranial hypertension (IIH), pseudotumor cerebri, executive function, neurocognition

## Abstract

Background: Idiopathic intracranial hypertension (IIH) is typically characterized by headaches and vision loss. However, neurocognitive deficits are also described. Our study aimed to test the influence of therapeutic lumbar puncture on the latter. Methods: A total of 15 patients with IIH were tested with a battery of neurocognitive tests at baseline and after therapeutic lumbar drainage. Hereby, Logical Memory of the Wechsler Memory Scale—Revised Edition (WMS-R), the California Verbal Learning Test Short Version (CVLT), alertness, selective attention, and word fluency were used. Changes in cognitive functioning in the course of CSF pressure lowering were analysed and compared with age, sex, and education-matched healthy controls. Results: Before intervention, scores of Logical Memory, the RWT, and the HADS-D were significantly lower in IIH patients compared to matched controls. After short-term normalization of CSF pressure, the RWT improved significantly. Additionally, significant positive correlations were found between headache intensity and subjective impairment, as well as between BMI and CSF opening pressure. Conclusions: Our findings confirm lower performance in terms of long-term verbal memory and word fluency compared to controls, as well as depressive symptoms in IIH patients. Significant improvement after short-term normalization of intracranial pressure by means of CSF drainage was seen only for word fluency. This indicates that short-term normalization of CSF pressure is not sufficient to normalize observed neurocognitive deficits.

## 1. Introduction

Idiopathic intracranial hypertension is a headache syndrome caused by elevated intracranial pressure (ICP) without evidence of an underlying structural lesion and is commonly seen in obese patients and in women of childbearing age [1]. Symptoms include headaches, progressive visual loss, and transient visual obscurations. Pathophysiological explanations include the malfunction of pressure regulation between blood vessels and CSF flow and, in recent studies, disturbances of the glymphatic system [2,3]. MR imaging of the brain and CSF analysis are usually normal, although indirect signs of increased intracranial pressure can be observed in a subset of patients. These include posterior eye globe flattening or empty sella [4]. Reported findings include papilledema, enlargement of the blind spot in visual field testing, and increased lumbar opening pressure > 25 cmH_2_O [5,6]. The mainstay of treatment is weight loss, mostly in combination with pharmacological options such as acetazolamide, and repeated CSF drainage for headache relief and improvements in vision [7]. In refractory cases, invasive options such as sinus stenting, bariatric surgery, fenestration of the optic nerve sheath, and shunting of CSF may be necessary [8].

Besides these common symptoms, recent research has highlighted the clinical relevance of cognitive impairments in IIH patients [9]. In the field of intracranial CSF flow disorders, this is evident in normal pressure hydrocephalus (NPH), particularly with regard to impairment in attention, memory, and executive and visuospatial function, and in the short-term improvement after CSF drainage by means of a spinal tap [10]. This improvement in cognitive function after a spinal tap is taken as a prognostic marker for the indication for ventricular shunting. In contrast, in IIH patients, cognitive impairment has been insufficiently studied, although case reports and small trials have shown impairment in cognition [11,12,13]. Sørensen et al. examined the cognition of 20 IIH patients for objective cognitive impairment (visual spatial memory, psychomotor speed, verbal memory, and learning). Five patients showed significant deficits in concentration, learning, and memory. Repeated testing after medication revealed improvements only in verbal tests and severe baseline impairments in psychomotor speed [11]. Kharkar et al. demonstrated short-term deficits in verbal memory in IIH patients. However, no significant deficits were found in visual memory [12]. In a more recent study by Wang and colleagues, IIH patients showed significantly poorer performance on several cognitive tests at baseline compared to the control group, including slower processing speed and weaker working memory, as well as more errors in eye movement tasks. These cognitive impairments persisted for over 6 months [13].

The main objective of our study was to understand and potentially evaluate interventions, e.g., lumbar drainage for cognitive impairment, in IIH patients in comparison to healthy age-matched controls with similar educational levels and to assess intragroup differences before and after normalizing CSF pressure by means of a lumbar tap.

## 2. Materials and Methods

### 2.1. Participants

This monocentric, prospective cohort study was performed at the Department of Neurology, University Hospital Munich. Patients were recruited between December 2017 and October 2018. The study was conducted in accordance with the Declaration of Helsinki and was approved by the ethics committee of the medical faculty of the Ludwig-Maximilians-University Munich (No. 17-340). All patients gave their written informed consent. The trial was also registered at the German Clinical Trial Register (DRKS) (DRKS00033278).

Fifteen patients between 18 and 70 years with an established diagnosis of IIH according to ICHD-3 were included. A new headache or at least twofold worsening of a pre-existing headache in patients diagnosed with IIH according to the Friedman criteria, with an increased intracranial pressure of at least >25 cmH_2_O and papilledema, was mandatory [6,14]. The presence of tinnitus was supportive. All patients had a clinical workup, including cranial MRI with venous angiography (to exclude hydrocephalus, mass, structural lesion, or sinus thrombosis), blood testing, and a spinal tap for the CSF analysis. Further inclusion criteria were stable medication for at least one week and no intake of any centrally acting agents immediately before testing. Individuals with concomitant pre-existing medical conditions that could also impact cognitive performance, such as severe psychiatric diseases, a history of acute drug or substance abuse, and a lack of ability to be assessed were excluded.

### 2.2. Procedure

**Figure 1:**
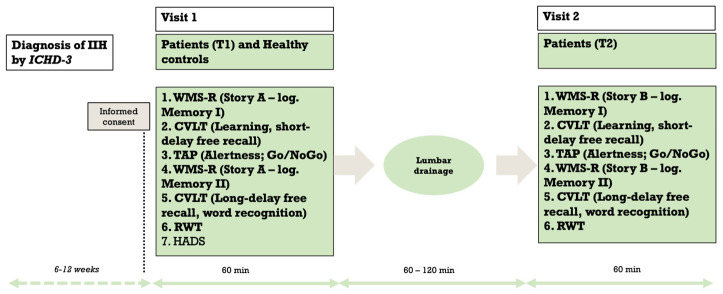
Workflow.

All participants were interviewed using a standardized questionnaire. BMI and a headache diary that tracked monthly headache days and intensity on a numerical rating range (NRS) from 0 to 10, where 10 represents maximum pain, were recorded at the time of the baseline medical examination. Following this, a standardized neurological examination was conducted by an experienced neurologist. Neuropsychological testing, lasting approximately 40–60 min, was performed in a quiet examination room before (Time, T1) and one to two hours after the spinal tap (Time, T2), as shown in Figure 1. Lumbar puncture for the CSF opening measurement was used as part of the indicated treatment and did not represent a study-specific procedure. The puncture was performed in a lying position, and spinal fluid was withdrawn until a target pressure of 20 cmH_2_O was reached. The opening pressure was measured with a calibrated manometer. On average, 20 to 30 mL of CSF was removed.

### 2.3. Neuropsychological Assessment

We used the Logical Memory of the Wechsler Memory Scale—Revised Edition (WMS-R), the California Verbal Learning Test Short Version (CVLT), alertness and selective attention from a larger computerized attention test battery, the Regensburg Word Fluency Test (RWT), and the Hospital Anxiety and Depression Scale (HADS) in our study population.

#### 2.3.1. Verbal Learning and Narrative Memory: Logical Memory of WMS-R

In this task, patients were asked to retell one story before and one after the lumbar puncture after it had been read aloud by an examiner. At first, the subtest Logical Memory I (immediate recall) was evaluated by having the patient recall Story A both immediately and after a delay of about 20 to 30 min (Logical Memory II (delayed recall)). After CSF drainage, Story B was used for Logical Memory I and II. A maximum of 25 points per story could be awarded, based on the accuracy of recall (for both immediate and delayed reproduction). Due to the lack of parallel versions, we used Story A before CSF puncture and Story B after CSF puncture in the study [15,16].

#### 2.3.2. Verbal Learning and Memory: CVLT Short Version

In addition, the California Verbal Learning Test Short Version (CVLT), which is designed for memory assessment, was administered to each patient. This test consists of a list of nine words divided into three categories. After each trial, participants were asked to recall as many words as possible in any order. After the fourth trial, the subjects were asked to count backwards from 100 to provide distraction. Subsequently, they were again asked to recall the words from memory without prior presentation (Short-Delay Free Recall). After 20 min, patients were again asked to recall the word list (Long-Delay Free Recall). Subsequently, patients were provided with the categories to aid in retrieving the corresponding words. At the end, after hearing 27 words read aloud, including the 9 initial words, participants were asked to decide whether the particular word was included in the category. The variables used were as follows: Trial 1, representing the number of words in the first trial as a measure of supra-span and immediate retention; Learning Sum, indicating the sum of words in learning trials 1–4 as an indicator of verbal learning performance; and finally, Long-Delay Free Recall [16,17,18].

#### 2.3.3. Alertness

We conducted two subtests from a larger computerized attention test battery (Test of Attentional Performance (TAP)): alertness and selective attention with response inhibition [16,19].

Phasic and Tonic Alertness: To evaluate tonic alertness, participants had to press a button as fast as possible after a cross appeared in white on a black screen. For phasic alertness, participants were required to press a button as fast as possible after a cross appeared in white on a black screen after a warning tone. We performed two runs without (tonic) and two with (phasic) warning sounds. The difference between the median RT for runs with and without audio warnings served as an indicator of phasic alertness. Go/NoGo: To investigate selective attention, individuals were required to respond as fast as possible, but only when a horizontal cross appeared, while refraining from responding to a vertical cross. The mean/median and standard deviation of reaction time, the number of correct responses (with and without warning sounds) (and in Go/NoGo: incorrect responses), the number of omissions (missing responses), outliers (reaction times greater than mean reaction time plus 2.35 × standard deviation of reaction times), and the number of anticipations (responses to audio warnings) were the output for each trial.

#### 2.3.4. Word Fluency

In the Regensburg Word Fluency Test (RWT), participants were instructed to list as many of the following words as possible in one minute each: any given first names, animals, and words beginning with “K” before the spinal tap; after the spinal tap, any first names, food items, and words beginning with “M”. The outcome variable was the total number of correctly produced words after subtracting errors across all three conditions. The validity of the RWT has been demonstrated for a variety of neurological disorders [16,20].

#### 2.3.5. Depression and Anxiety

The Hospital Anxiety and Depression Scale (HADS) was used to assess anxiety (HADS-A) and depression (HADS-D) symptoms. Scores of 8 points or above for each scale were seen as suspicious, whereas scores equal to or above 11 points indicated an abnormal result for depression and anxiety [21].

### 2.4. Statistical Analysis

Data were collected at two different time points on the same day: T1: baseline before therapeutic lumbar puncture; T2: 1 to 2 h after therapeutic lumbar puncture. All data obtained are expressed as mean ± SD. Statistical significance was considered at a *p*-value of <0.05. SPSS (Version 25, IBM, SPSS Inc., Chicago, IL, USA) was used for the statistical analysis. Descriptive statistics were performed to analyse frequencies, and mean and standard deviations (±SD) were used for the metric data. In normally distributed demographic data, we used an independent *t*-test. The Wilcoxon test was used for the comparison of paired data and the Mann–Whitney U test was used for comparisons between patients and controls. We additionally performed the post hoc Bonferroni correction for multiple testing. Correlations between non-normally distributed data were tested using Spearman’s correlation. The distribution of the data was tested with the Kolmogorov–Smirnov test. Plausible outliers were removed if they were 1.5 IQRs (interquartile ranges) above the third or below the first quartile. The effect sizes of significant results were calculated using Pearson’s correlation coefficient (r).

## 3. Results

### 3.1. Demographic Data

The IIH group included 15 patients (3 male, 12 female, with a mean age of 38.6 ± 12.9 years). The control group consisted of 15 age, sex, and education-matched subjects (3 male, 12 female, with a mean age of 38.5 ± 12.5 years). None of the healthy controls fulfilled the criteria of IIH according to ICHD3 [14], nor did they have any other frequent primary headaches. Some participants in the control group had a history of sporadic headaches, such as headaches associated with influenza or following alcohol exposure. The only significant demographic difference was body mass index (BMI), which was significantly higher in the IIH patients compared to the healthy controls (36 ± 6.2 kg/m^2^ vs. 24.8 ± 5.3 kg/m^2^; unpaired *t*-test *p* ≤ 0.01). Table 1 presents the full demographic data.

### 3.2. Clinical Characteristics

At the time of testing, headaches as an ongoing symptom were present in 60% of the patients. We obtained ratings of maximal headache pain with an NRS at T1 and T2 from each group. The mean NRS in the IIH cohort was 3.5 ± 3.6. Other symptoms of the patient population included visual disturbances (visual field, visual loss) in 11 patients (73.3%) and hearing disturbances (tinnitus) in 10 patients (66.6%). Subjective cognitive impairments were described by 11 patients (73.3%).

The mean cerebrospinal fluid (CSF) opening pressure in a lying position was 33.0 ± 7.2 cmH_2_O. A total of 26.7% had an opening pressure below 30 cmH_2_O. Among patients with headaches (n = 9), the mean lumbar opening pressure reached 32.1 ± 8.1 cmH_2_O, whereas in individuals without a current headache (n = 6), the mean lumbar opening pressure was 34.3 ± 6.2 cmH_2_O. However, the difference between lumbar pressure and headaches did not reach statistical significance (*z* = −0.30; *p* = 0.77) (clinical data in Table 2). Furthermore, no patient showed evidence of secondary intracranial hypertension such as sinus vein thrombosis, and the analysis of CSF was unremarkable in all cases.

A higher BMI significantly correlated with higher lumbar opening pressure (*r* = 0.676, *p* < 0.001, n = 15). Additionally, headache intensity showed a significant correlation with subjective impairment (*r* = 0.715, *p* = 0.001, n = 15). However, no significant correlations were found between BMI and headache intensity, between lumbar opening pressure and headache intensity, or between subjective impairment and HADS score (HADS-A and HADS-D).

### 3.3. Neuropsychological Assessment in the Total Sample

#### 3.3.1. Comparison between IIH Patients and Healthy Controls at Baseline (T1)

In contrast to the healthy controls, patients showed significantly lower scores in delayed verbal recall (Logical Memory II, WMS-R, 10.7 ± 5.0 vs. 14.5 ± 2.5, *z* = 2.19, *p* = 0.029), exhibiting large effect sizes (Pearson’s *r* = 0.57). The same applied to word fluency (RWT), where the IIH patients underperformed compared to the controls (54.1 ± 14.0 vs. 71.9 ± 14.4 *z* = 3.03, *p* = 0.002), with a strong effect size (Pearson’s *r* = 0.78). Additionally, HADS values for depression (HADS-D) were higher in the IIH cohort (5.6 ± 4.3 vs. 1.9 ± 2.2, *z* = 2.69, *p* = 0.007), again with a large effect size (Pearson’s *r* = 0.69). Linear regression revealed no associations between HADS-D scores and Logical Memory II or tonic alertness. However, each point increase in HADS-D score was associated with a 2-point reduction in the RWT (F = 8.67, adjusted R^2^ = 0.35, *p* = 0.011). In summary, at baseline (i.e., before lumbar puncture), scores for Logical Memory II (WMS-R), the RWT, and the HADS-D were lower in IIH patients compared to controls. No significant differences between groups were observed in a second verbal memory task (CVLT scores), in alertness and selective attention (TAP), or in psychiatric symptoms (HADS-A). The SD and mean data for intergroup comparisons are summarized in Table 3.

#### 3.3.2. Comparison in IIH Patients before (T1) and after Lumbar Puncture (T2)

Regarding intragroup comparisons before and after lumbar puncture, only the RWT improved significantly (54.1 ± 14.0 vs. 62.6 ± 14.9, *z* = −2.16, *p* = 0.031), indicating a large effect (Pearson’s *r* = 0.56). However, after performing the Bonferroni correction for multiple testing, these results missed statistical significance at an adjusted *p* ≤ 0.01. The SD and mean data are summarized in Table 4.

## 4. Discussion

Neurocognitive impairment is frequently reported by patients with idiopathic intracranial hypertension (IIH). Another well-known disorder of CSF circulation is normal pressure hydrocephalus (NPH), with neurocognitive dysfunction as a cardinal symptom of the clinical–diagnostic Hakim triad and significant improvement in it after CSF tapping [10].

According to the baseline characteristics of the patient cohort, mean age, female predominance, and BMI were consistent with previously published studies [5]. Moreover, our findings revealed a positive correlation of CSF opening pressure and BMI, aligning with prior trials [22,23]. However, there was no correlation of lumbar opening pressure with headache intensity in our cohort. This adds to the ongoing debate regarding the association of opening pressure and headache [24]. There was also no association between headache intensity and BMI, which supports the hypothesis of a dissociation between headache and ICP. Potential independent mechanisms between headache and ICP, such as Calcitonin gene-related peptide-dependent headache, have been discussed quite recently [3]. Notably, the HADS-D scores were significantly higher within the IIH cohort. This can be explained by the association of IIH with socioeconomic and psychological difficulties [25]. Furthermore, we observed that HADS-A and HADS-D scores significantly correlated with each other, which could be expected since anxiety and depression have a bivalent relation.

To evaluate cognitive function at baseline in IIH patients, we first applied intergroup comparisons. Regarding Logical Memory of the WMS-R, our results confirm reduced performance in long-term verbal memory recall (Logical Memory II), compared to the healthy controls. This is consistent with earlier studies [12,26]. However, Kharkar and colleagues also showed deficits in short-term verbal memory recall (Logical Memory I) [12]. Regarding CVLT scores, no significant differences between groups were found. However, impairment in Long-Delay Free Recall would be expected based on previous findings in Logical Memory II of the WMS-R. This inconsistency can be explained by the distinction between the CVLT and recalling individual words in contrast to Logical Memory, which is about retaining more coherent information. In our study, there was no difference between tonic and phasic alertness between groups, similar to another trial in which only 2% of IIH patients underscored in attention tasks [27]. Finally, the results of the RWT showed significant impairment in word fluency in IIH patients, which is consistent with earlier data [28]. It is noteworthy that word fluency correlated negatively with an elevated HADS-D score. In a meta-analytic review, decreased word fluency in depression was attributed more to a global deficit than to executive dysfunction [29]. However, word fluency deficits can also be interpreted as nonspecific avolition. In summary, our findings indicate an impairment of long-term verbal memory (WMS-R), word fluency (RWT), and depressive symptoms (HADS-D) in IIH patients at baseline when compared with healthy controls.

To assess the effect of lumbar puncture on cognition in IIH patients, we compared the aforementioned batteries at baseline and post-intervention. Intragroup comparisons only revealed a significant improvement in word fluency, which may be related to frontal cortex dysfunction, similar to NPH [30,31]. However, this effect was non-significant after performing the Bonferroni correction. Performance in other neuropsychological tasks did not change significantly after CSF puncture, coinciding with previous work from Fermo and Yri and colleagues [27,32]. In contrast, Grech et al. showed that neurocognitive deficits, particularly in the areas of acute attention and verbal short-term memory, improve in the long term as a result of a lumbar puncture [9]. In conclusion, our study revealed poorer outcomes in IIH patients compared to healthy controls (for verbal memory, word fluency, and depression and anxiety symptoms). Intragroup comparisons only showed significant improvements for word fluency in IIH patients after lumbar puncture, but no performance improvement could be shown regarding any other cognitive functions. Therefore, overall, no positive effect on cognition after lumbar puncture could be shown.

### Limitations

There are several limitations to consider in our study. Firstly, the relatively small number of participants is an important limitation. However, our IIH and control groups were homogeneous for important parameters (i.e., age, education) and our group sizes were considerably larger compared with most previously published studies on this topic. Additionally, the analysis of intraindividual changes at two time points mitigates this limitation. Furthermore, the high proportion of IIH patients with comorbid depressive symptoms may have an important impact on cognitive test performance. However, the HADS-D score was only mildly to moderately increased, indicating no severe depression (HADS-D > 8) in our IIH patients. Furthermore, there is also evidence that there is no statistically significant relationship between depression and neurocognition in IIH [13]. A further important factor to consider is that most IIH patients use acetazolamide as a prophylactic medication. Detailed effects of that drug on neurocognition are not known but may bias our findings. Otherwise, the medication should not affect the pre- and post-lumbar puncture comparison in the patients. Finally, the short time interval between the lumbar puncture for CSF tapping and the second neurocognitive test is a limiting factor.

## 5. Conclusions

Despite limitations, our findings strongly suggest subtle neuropsychological impairments in verbal long-term memory, compared to healthy controls, which do not normalize as a short-term effect after the lowering of intracranial hypertension to normal values. Presumably, different secondary factors from intracranial hypertension per se might contribute to these persisting cognitive deficits in IIH. Possible explanations discussed include venous stasis or deficits in the clearage of cytotoxic substances. Further research is needed to understand the pathophysiology of neurocognitive deficits in IIH and to evaluate associations between cognitive deficits and functional impairment, as the latter may be a major contributing factor in IIH patients.

## Figures and Tables

**Table 1:** Demographic data of total population.

	IIH Patients (n =15)	Healthy Controls (n = 15)
Female (%)	12 (80)	12 (80)
Male (%)	3 (20)	3 (20)
Age (years)	38.6 ± 12.9	38.5 ± 12.5
**BMI (kg/m^2^)**	**36.0 ± 6.2**	**24.8 ± 5.3**
Education		
Lower secondary school certificate (%)	4 (26.7)	4 (26.7)
Intermediate school-leaving certificate (O level) (%)	7 (46.7)	7 (46.7)
Higher education entrance qualification (A levels) (%)	4 (26.7)	4 (26.7)

Significant changes and effect sizes are marked in bold.

**Table 2:** Clinical data of patient cohort.

Clinical Data	IIH Patients (n = 15)
Lumbar opening pressure (cmH_2_O)	33 ± 7.2
Lumbar opening pressure ≥ 25 cmH_2_O (n) (%)	13 (86.6%)
Lumbar opening pressure ≥ 30 cmH_2_O (n) (%)	11 (73.3%)
Subjective impairment (NRS)	6.3 ± 3.2
Headache at time of testing (n) (%)	9 (60.0%)
Headache intensity (NRS)	**3.5 ± 3.6**
Cognitive impairment (n) (%)	**11 (73.3%)**
Subjective improvement in cognitive impairment after lumbar puncture (n) (%)	11 (73.3%)
Visual disturbances (n) (%)	11 (73.3%)
Tinnitus (n) (%)	10 (66.6%)
Medication for IIH (n) (%)	13 (86.6%)
Acetazolamide (n) (%)	12 (80.0%)
Topiramate (n) (%)	1 (6.6%)
Employed at time of testing (n) (%)	15 (100.0%)

Significant changes and effect sizes are marked in bold.

**Table 3:** Results of IIH patients and healthy controls at baseline.

	IIH Patients (n = 15) (Mean ± SD)	Healthy Controls (n = 15) (Mean ± SD)	*p*-Value	Wilcoxon *z*	Effect Size Pearson’s *r*
WMS-R					
Logical Memory I	12.5 ± 4.4	15.1 ± 2.8	0.13		
Logical Memory II	10.7 ± 5.0	14.5 ± 2.5	**0.029**	2.19	**0.57**
CVLT					
Trial 1	5.2 ± 1.5	5.6 ± 1.4	0.51		
Learning Sum	27.4 ± 5.4	29.9 ± 4.1	0.20		
Long-Delay Free Recall	6.8 ± 2.1	7.8 ± 1.5	0.21		
TAP: Alertness					
Median reaction time without warning tone	268.3 ± 36.2	245.9 ± 36.6	0.12		
Median reaction time with warning tone	266.9 ± 39.7	250.53 ± 37.8	0.29		
Phasic alertness	−0.01 ± 0.2	−0.01 ± 0.1	0.42		
TAP: Go/NoGo					
Incorrect responses	1.1 ± 1.7	0.9 ± 1.2	0.95		
Median reaction time	423.8 ± 63.1	426.8 ± 82.8	0.91		
RWT	54.1 ± 14.0	71.9 ± 14.4	**0.002**	3.03	**0.78**
HADS-D	5.6 ± 4.3	1.9 ± 2.2	**0.007**	2.69	**0.69**
HADS-A	7.2 ± 4.5	4.4 ± 3.2	0.08		
HADS-G	12.8 ± 8.5	6.3 ± 4.7	**0.033**		

Significant changes and effect sizes are marked in bold.

**Table 4:** Results of IIH patients before and after therapeutical lumbar puncture.

IIH-Patients (n = 15)	T1 (Mean ± SD)	T2 (Mean ± SD)	*p*-Value	Wilcoxon *z*	Effect Size Pearson’s *r*
WMS-R					
Logical Memory I	12.5 ± 4.4	12.3 ± 4.4	0.83		
Logical Memory II	10.7 ± 5.0	11.6 ± 4.8	0.42		
CVLT					
Trial 1	5.2 ± 1.5	5.3 ± 1.7	0.87		
Learning Sum	27.4 ± 5.4	27.9 ± 5.8	0.67		
Long-Delay Free Recall	6.8 ± 2.1	6.7 ± 3.0	0.79		
TAP: Alertness					
Median reaction time without warning tone	268.3 ± 36.2	277.6 ± 36.6	0.13		
Median reaction time with warning tone	266.9 ± 39.7	268.5 ± 38.5	0.48		
Phasic alertness	−0.01 ± 0.2	0.01 ± 0.1	0.17		
TAP: Go/NoGo					
Incorrect responses	1.1 ± 1.7	1.9 ± 2.3	0.08		
Median reaction time	423.8 ± 63.1	436.0 ± 80.9	0.33		
RWT	54.1 ± 14.0	62.6 ± 14.9	**0.031**	**−2.16**	**0.56**

Significant changes and effect sizes are marked in bold.

## Data Availability

The data are contained within the article.
